# Prevalence of depressive symptoms in pregnant and postnatal HIV-positive women in Ukraine: a cross-sectional survey

**DOI:** 10.1186/s12978-016-0150-z

**Published:** 2016-03-22

**Authors:** Heather Bailey, Ruslan Malyuta, Igor Semenenko, Claire L Townsend, Mario Cortina-Borja, Claire Thorne, for the Ukraine European Collaborative Study in EuroCoord

**Affiliations:** UCL Institute of Child Health, University College London, London, UK; Perinatal Prevention of AIDS Initiative, Odessa, Ukraine; Population, Policy and Practice Programme, UCL Institute of Child Health, University College London, London, UK

**Keywords:** Depression, HIV infection, Eastern Europe, Pregnancy, Postpartum period, Antiretroviral therapy, Prevention of mother-to-child transmission, Ukraine

## Abstract

**Background:**

Perinatal depression among HIV-positive women has negative implications for HIV-related and other maternal and infant outcomes. The aim of this study was to investigate the burden and correlates of perinatal depression among HIV-positive women in Ukraine, a lower middle income country with one of the largest HIV-positive populations in Europe.

**Methods:**

Cross-sectional surveys nested within the Ukraine European Collaborative Study were conducted of HIV-positive women at delivery and between 1 and 12 months postpartum. Depressive symptoms in the previous month were assessed using a self-report screening tool. Other data collected included demographics, antiretroviral therapy (ART)-related self-efficacy, and perceptions of risks/benefits of interventions to prevent mother-to-child transmission (PMTCT). Characteristics of women with and without a positive depression screening test result were compared using Fisher’s exact test and χ^2^ test for categorical variables.

**Results:**

A quarter (27 % (49/180) antenatally and 25 % (57/228) postnatally) of participants screened positive for depressive symptoms. Antenatal risk factors were living alone (58 % (7/12) vs 25 % (42/167) *p* = 0.02), being somewhat/terribly bothered by ART side effects (40 % (17/43) vs 23 % (30/129) not /only slightly bothered, *p* = 0.05) and having lower ART-related self-efficacy (43 % (12/28) vs 23 % (25/110) with higher self-efficacy, *p* = 0.05). Postnatally, single mothers were more likely to screen positive (44 % (20/45) vs 21 % (18/84) of cohabiting and 19 % (19/99) of married women, *p* < 0.01) as were those unsure of the effectiveness of neonatal prophylaxis (40 % (20/45) vs 18 % (28/154) sure of effectiveness, *p* < 0.01), those worried that neonatal prophylaxis could harm the baby (30 % (44/146) vs 14 % (10/73) not worried *p* < 0.01) and those not confident to ask for help with taking ART (48 % (11/23) vs 27 % (10/37) fairly confident and 15 % (4/26) confident that they could do this). Of women who reported wanting help for their depressive symptoms, 82 % (37/45) postnatally but only 31 % (12/39) antenatally were already accessing peer counselling, treatment adherence programmes, support groups or social services.

**Conclusions:**

A quarter of women screened positive for depression. Results highlight the need for proactive strategies to identify depressive symptoms, and an unmet need for provision of mental health support in the perinatal period for HIV-positive women in Ukraine.

## Background

In Ukraine, a lower middle income country with one of the most severe HIV epidemics in Europe, an estimated 95,000 women are living with HIV and around 4000 infants are born to women living with HIV each year [1, 2]. In the general population of Ukraine, the WHO World Mental Health Survey (2001-03) found the 12-month prevalence of mood disorder to be 9.1 % (the second highest of 14 countries surveyed) [3], and the 12-month prevalence of major depressive disorder among women to be 11.3 %, with unmarried women and those with low educational or socioeconomic status at increased risk [4]. Little information is available on the mental health of HIV-positive women living in Eastern Europe, but studies from other settings (mainly North America and Africa) have shown high prevalence of perinatal depression in this patient group linked with a range of factors including socioeconomic adversity, intimate partner violence and HIV-related stigma [5]. Pregnancy and the postnatal period are times of heightened depression risk regardless of HIV status [6, 7]. This risk may be further compounded in HIV-positive women by inequalities in access to support and services and by the HIV diagnosis itself [8, 9] which, for about 60 % of HIV-positive women delivering in Ukraine each year, has occurred during that pregnancy [10].

Adverse consequences of maternal depression for women living with HIV and their children may include a negative impact on uptake of and adherence to interventions to prevent mother-to-child transmission (PMTCT) [11, 12] and on maternal HIV disease outcomes beyond pregnancy [13, 14], as well as increased risk of preterm delivery [15] and of developmental and behavioural problems in the child [16, 17]. Screening for depression is not currently part of routine perinatal or HIV care in Ukraine, and mental health services – historically delivered in secondary care – are highly stigmatised [18]. The aim of this analysis was to investigate the burden and correlates of perinatal depression among women living with HIV in Ukraine.

## Methods

### Design, setting and participants

Two cross-sectional surveys (hereafter referred to as the ‘antenatal survey’ and ‘postnatal survey’) were conducted among HIV-positive women attending for obstetric and/or HIV care in Ukraine between July to December 2011, with extension to April 2012 for some sites. The primary objective of the surveys was to measure adherence to antiretroviral therapy (ART) in pregnancy and postnatally [19]. The current study is a secondary analysis of survey data, to investigate prevalence of and factors associated with depressive symptoms in this study population.

The antenatal survey was conducted among HIV-positive women giving birth at one of three participating maternity hospitals in Kiev, Odessa and Simferopol and having received ART for at least the last four weeks of their pregnancy (i.e. engaged in HIV care in pregnancy). The survey was completed during the hospital stay following delivery (typically of ≥3 days), with retrospective self-report of depressive symptoms experienced during the last month of pregnancy. Clinicians at study sites identified eligible women, provided them with written and verbal information on the study, and gave them an anonymous paper-based questionnaire (in Russian) to complete if they consented to participate. The postnatal survey was conducted among HIV-positive women attending one of six participating regional HIV/AIDS centres (in Kiev, Odessa, Mykolaiv, Donetsk, Kriviy Rig and Simferopol) between 1 and 12 months postpartum. Women were eligible regardless of treatment history, and were identified and invited to participate by their clinician during the course of routine infant follow-up and ongoing HIV care, with informed consent procedures as for the antenatal survey.

These surveys were nested within the infrastructure of the European Collaborative Study (ECS) in EuroCoord (www.eurocoord.net), a multi-site observational cohort study of HIV-infected pregnant women and their infants enrolling women in Ukraine since 2000 [10], however ECS enrolment was not a pre-requisite for survey participation. The anonymous individual patient survey data were matched to patient records in the ECS database using four variables (maternal date of birth, date of delivery, infant sex and centre), to give additional information on clinical characteristics.

### Depression screening tool and other measures

In the antenatal and postnatal surveys, the Patient Health Questionnaire (PHQ)-2 Screening Questions from the Primary Care Evaluation of Mental Disorders [20] were used to identify depressive symptoms (i.e. anhedonia and low mood) over the past month. These two questions are as follows: ‘During the past month, have you often been bothered by feeling down, depressed or hopeless?’, and ‘During the past month, have you often been bothered by little interest or pleasure in doing things?’ Each question has a yes/no response. A participant was considered to have a positive depression screening test result if she responded ‘yes’ to both of these questions, or if she answered ‘yes’ to either one and also responded positively to the follow-on question ‘Is this something you feel you need or want help with?’ (i.e. responded ‘yes’ or ‘yes but not today’, rather than the remaining option of ‘no’) [21]. ART-related self-efficacy was measured using five questions adapted from the AIDS Clinical Trials Group Center for AIDS Prevention Studies HIV Treatment Adherence Self-Efficacy Scale [22]. These questions asked about her level of confidence to perform various behaviours over the last four weeks, for example, to keep taking medication even if side effects began to interfere with her daily activities, and to keep taking medication even if in front of people who were not aware of her HIV status. The questionnaire also included questions about health behaviours, HIV status disclosure, experience of ART side effect and help-seeking self-efficacy [22]. Perceptions of the risks and benefits of ART for PMTCT were assessed using questions adapted from the NIAIDS Adult AIDS Clinical Trials Group supplemental antepartum adherence questionnaire [23]; women were asked how sure they felt that antenatal ART (antenatal survey) or neonatal prophylaxis (postnatal survey) were effective for PMTCT and how worried they were about these interventions harming their baby.

### Statistical analysis

Survey data from 10 respondents (five in the antenatal survey and five in the postnatal survey) who did not complete the two depression screening questions on anhedonia and low mood were excluded from the analysis. Seven women in the antenatal survey and ten in the postnatal survey who self-reported anhedonia or low mood but did not answer the follow-on question about need for help were categorised as screen-negative in the main results, and screen-positive in a sensitivity analysis. The responses of 11 women who participated in the antenatal survey and later in the postnatal survey were included in results for both groups. Characteristics of women screening positive and negative for depression were compared using the Fisher’s exact test and the χ^2^test for categorical variables; unless otherwise stated, *p*-values were obtained using two-sided Fisher’s exact tests. A probability of 0.05 was used to define statistical significance. Data were managed using REDCap electronic data capture tools hosted at University College London Institute of Child Health [24]. Statistical analyses were performed using STATA version 12.1 (StataCorp, LP, College Station, Texas, USA).

### Ethics, consent and permissions

Consent was given verbally, reflecting the anonymous nature of the study, and return of a completed questionnaire was taken as documentation and evidence of a woman’s consent to participate. As part of the survey protocol, each completed questionnaire was reviewed by the recruiting clinician in order to identify women screening positive for depressive symptoms who required referral for support. This was explained in the patient information sheet. Ethical approval for these surveys was obtained from the UCL Research Ethics Committee (3061/001), in addition to institutional approvals from participating sites. The ECS has approval from the Great Ormond Street Hospital for Children NHS Trust/Institute of Child Health Research Ethics Committee.

## Results

There were 180 antenatal survey respondents, giving an estimated participation rate in the main six-month survey period of 39-49 % [19]. Of the total 228 postnatal survey respondents, 137 took part during July to December 2011 at five HIV/AIDS centres enrolling also into a postnatal cohort within the ECS; using denominator data from these sites, the participation rate for this period was estimated at 35 % (137/396). This estimate considered only women attending the HIV/AIDS centre for their first postnatal visit, and not those returning for follow-up.

Overall, across both surveys, median age was 28.0 years (IQR 24.8-31.3), 19 % (79/408) of women were single and 12 % (46/389) reported an illicit drug use history (not including marijuana). Thirty percent (117/386) reported that their pregnancy was unplanned. By survey completion, 91 % (163/179) of antenatal and 95 % (210/222) of postnatal participants had disclosed their HIV status to at least one person; among the 79 % (323/408) who were married or cohabiting, 87 % (282/323) had disclosed to their partner. Among antenatal respondents and the 44 % (101/228) of postnatal respondents currently on ART, a quarter (66/271) reported being somewhat or terribly bothered by ART side effects, while 20 % (28/138) antenatally and 17 % (11/66) postnatally reported low ART-related self-efficacy (i.e. unable to do one or more of five ART-related activities). The proportion of women already using one or more of the support services available as part of HIV care (peer counselling, treatment adherence programmes, support groups and social services) was 78 % (78/100) in the postnatal group restricted to women enrolled in Kiev, Odessa and Simferopol, but only 32 % (58/180) in the antenatal group enrolled in the same cities (χ^2^ = 53.93 *p* < 0.01); the majority of these antenatal participants were using social services only, while the postnatal participants were more commonly using multiple services including support groups and peer counselling (Table 1).

**Table 1:** Characteristics of survey participants

	Antenatal Survey (*n*=180)	Postnatal Survey (*n*=228)
Median age at participation (IQR)	27.4 years (24.8, 30.9)	28.3 years (24.8, 31.9)
Marital status		
Married	96/180 (53 %)	99/228 (43 %)
Cohabiting	50/180 (28 %)	84/228 (37 %)
Single	34/180 (19 %)	45/228 (20 %)
Living as only adult in household	12/179 (7 %)	28/225 (12 %)
Disclosure of HIV status		
To husband/partner or family or friend(s)	162/178 (91 %)	210/222 (95 %)
To no one at all	16/178 (9 %)	12/222 (5 %)
Smoking, alcohol and drug use^a^		
Current smoker	47/178 (26 %)	74/225 (33 %)
Current alcohol use	15/179 (8 %)	18/224 (8 %)
Ever used marijuana	25/178 (14 %)	27/228 (12 %)
Ever used illicit drugs other than marijuana	23/174 (13 %)	23/215 (11 %)
Use of support services^a^		
Currently using support group	10/180 (5 %)	33/228 (14 %)
Currently using peer counselling	9/180 (5 %)	85/228 (37 %)
Currently using social services	39/180 (22 %)	82/228 (36 %)
Currently using adherence programme	4/180 (2 %)	43/228 (19 %)
On ART for 4 weeks preceding survey^b^	180/180 (100 %)	101/228 (44 %)

^**a**^Groups not mutually exclusive

^b^Receipt of antenatal ART was one of the eligibility criteria for participation in the antenatal survey. Fewer women were on ART in the postnatal group because Ukraine's national policy at the time of these surveys was based on WHO Option B, with pregnant women who did not yet have treatment indications for their own health stopping ART at delivery

Around half in each survey group (93/180 antenatal survey participants and 109/228 postnatal participants) had matched ECS data available; in this sub-group, 57 % (52/92) and 60 % (65/109) respectively were diagnosed as HIV-positive during their recent pregnancy, and 22 % (18/83) and 16 % (17/104) had WHO stage 3-4 disease (χ^2^ = 0.20 *p* = 0.66). However, among women in the postnatal survey who had remained on ART after delivery, 39 % (16/41) had WHO stage 3-4 disease vs. only 1/66 of those not on ART postpartum, reflecting indications for treatment outside pregnancy within clinical guidelines at that time.

**Fig. 1:**
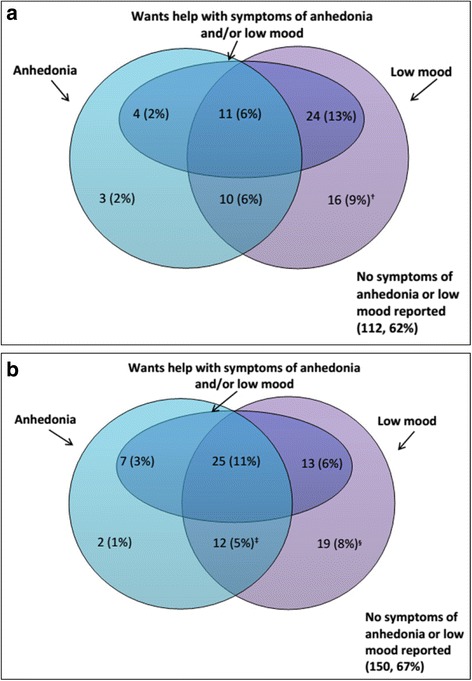
Responses to three depression screening questions among (**a**) 180 antenatal (**b**) 228 postnatal survey participants. The overlapping segments indicate the women who screened positive (i.e. who reported anhedonia and low mood, or at least one of these symptoms in addition to wanting help). A response to the question on wanting help was missing for ^†^7 of 16 women who reported only low mood in the antenatal survey, ^‡^1 of 12 women who reported both low mood and anhedonia in the postnatal survey and ^§^9 of 19 women who reported only low mood in the postnatal survey

Overall, 27 % (95 % CI 21-34) (49/180) of antenatal survey respondents and 25 % (95 % CI 20-31) (57/228) of postnatal survey respondents had a positive depression screening test. Anhedonia was reported by 16 % (28/180) antenatally and 20 % (46/228) postnatally and low mood by 34 % (61/180) and 30 % (69/228) (Fig. 1a and 1b). Among the 68 (37 %) antenatal survey participants who reported one or both symptoms, 61 answered the follow-on question ‘is this something you feel you need or want help with?’, of whom 39 % (*n* = 24) responded ‘yes’ and a further 36 % (*n* = 15) responded ‘yes, but not today’; in the postnatal survey, these proportions were 32 % (22/68) and 34 % (23/68) respectively. Of the 21 women reporting both symptoms in the antenatal survey, 52 % [11] reported wanting help while postnatally this proportion was 68 % (25/37). If the seven women in the antenatal survey and the 10 in the postnatal survey who reported anhedonia and/or low mood but omitted the ‘help’ question had all reported needing /wanting help, the overall prevalence of screen positive results would have increased from 27 % to 31 % (56/180) in the antenatal survey and from 25 % to 29 % (66/228) in the postnatal survey.

Factors associated with a positive depression screening test are shown in Table 2. Women living alone (antenatal survey) or without a cohabiting partner (postnatal survey) were at increased risk, as were those with low ART-related self-efficacy (statistically significant in the antenatal survey only). Postnatally, 45 % (5/11) vs. 25 % (14/55) of women with low vs. high self-efficacy screened positive for depression (*p* = 0.17). There was some indication of an association between severity of ART side effects and depressive symptoms during pregnancy (Table 2, *p* = 0.05) but not postnatally among the subgroup of 99 women on ART (26 % (7/27) of the screen positive women reported being somewhat /terribly bothered by side effects vs 22 % (16/72) of the screen negative, *p* = 0.79). Antenatally, depression was more common among the small group unsure of the effectiveness of ART for PMTCT although this did not reach statistical significance (Table 2, *p* = 0.06), while postnatally it was more common among the 29 % (63/217) unsure of the effectiveness of neonatal prophylaxis, and the two-thirds (146/219) who worried that prophylaxis might harm their baby (Table 2). In the postnatal survey, there was also a higher prevalence of screen positive results among women who felt unable to ask for help with their medication (Table 2, *p* = 0.05).

**Table 2:** Factors associated with a positive depression screening test

	Positive depression screening test	Fisher’s exact test
*Antenatal survey*		
Living as only adult in household?		
No	25 % (42/167)	*p*=0.02
Yes	58 % (7/12)	
Severity of ART side effects		
Not bothered by side effects or only slightly	23 % (30/129)	*p*=0.05
Somewhat or terribly bothered by side effects	40 % (17/43)	
Level of confidence that antenatal ART is effective for PMTCT		
Completely/fairly sure that it is effective	25 % (43/169)	*p*=0.06
Not at all sure that it is effective	56 % (5/9)	
Self-efficacy score for integration of ART into daily life		
≥5 (higher self-efficacy)	23 % (25/110)	*p*=0.05
<5 (lower self-efficacy)	43 % (12/28)	
*Postnatal survey*		
Marital status		
Married	19 % (19/99)	*p*<0.01
Co-habiting	21 % (18/84)	
Single	44 % (20/45)	
Can neonatal prophylaxis help prevent MTCT?		
Yes	18 % (28/154)	*p*<0.01
Not sure/No	40 % (25/63)	
Ever worried that neonatal prophylaxis could harm baby		
Yes/a little	30 % (44/146)	*p*<0.01
No	14 % (10/73)	
Self-rated ability to ask for support with taking medication^a^		
Confident I could do	15 % (4/26)	*p*=0.05
Fairly confident could do	27 % (10/37)	
Could not do	48 % (11/23)	

ART – antiretroviral therapy; MTCT – mother-to-child transmission

^**a**^Available for 86 of the 101 women on ART at time of postnatal survey completion

Of the 228 women completing the postnatal survey, timing of survey completion was available for 225; 53 % (*n* = 120) participated at between 1 and 6 months postpartum and 47 % (n = 105) at between 6 and 12 months. There was no difference in timing of survey completion by depression screening test result (those screening positive completed the survey at a median of 167 days after delivery vs 172 days for those screening negative, Wilcoxon rank-sum test *p* = 0.79). The proportion using at least one support service was similar overall by depression screening test result (antenatally, 31 % (15/49) of screen positive women vs 33 % (43/131) of screen negative, χ^2^ = 0.08, *p* = 0.78; postnatally, 81 % (46/57) and 70 % (120/171) respectively, χ^2^ = 2.39, *p* = 0.12). Of those who screened positive and reported wanting help for their depressive symptoms, 80 % (36/45) in the postnatal group were already using at least one support service vs. only 23 % (9/39) in the antenatal group (*p* < 0.01).

In the antenatal survey, prevalence of screen positive results for depression was 25 % (31/126) among those who reported that their pregnancy was planned vs 35 % (17/49) among those with unplanned pregnancies (χ^2^ = 1.80, *p* = 0.18) while postnatally these proportions were 24 % (35/143) and 26 % (18/68) respectively (χ^2^ = 0.10, *p* = 0.76). Antenatal participants diagnosed as HIV-positive during their most recent pregnancy were no more likely to screen positive than those diagnosed before pregnancy (31 % (16/52) and 28 % (11/40) respectively, *p* = 0.82), however in the postnatal group there was some indication of an association (31 % (20/65) diagnosed during most recent pregnancy screened positive vs 16 % (7/44) diagnosed prior to pregnancy) although this did not reach statistical significance (*p* = 0.11). Among married or cohabiting women with disclosure data available (145 in the antenatal survey and 177 in the postnatal survey), there was no association between disclosure of HIV status to partner and positive screening test (antenatally, 29 % (34/119) of those who had disclosed screened positive vs 15 % (4/26) of those who had not disclosed (*p* = 0.22) while postnatally these proportions were 21 % (34/162) and 20 % (3/15), *p* = 1.00). Prevalence of screen positive results was 34 % (22/65) among women with WHO stage 1-2 disease vs. 11 % (2/18) among those with WHO stage 3-4 in the antenatal survey (*p* = 0.08) and 24 % (21/87) vs. 29 % (5/17) respectively in the postnatal survey (*p* = 0.76).

## Discussion

One in four women living with HIV in this study screened positive for depression, with a similar prevalence in antenatal and postnatal surveys. Women who had poor social support, doubts about the safety and effectiveness of interventions for PMTCT, lower ART-related self-efficacy and, during pregnancy, more severe self-reported ART side effects, were more likely to screen positive. Our results highlight a need for strategies to identify and support women with depressive symptoms among this high-risk population, and for specific counselling to address concerns and problems around use of ART for PMTCT and treatment.

The self-report tool used in this study, although used as part of routine perinatal care in the UK [25, 26], has not yet been validated in Russian and assessment of its sensitivity and specificity in this population was outside of the scope of this study; we are therefore not able to make conclusions about how well the screening tool differentiated between women with and without symptoms meeting diagnostic criteria for depression . It did however have a high level of acceptance among participants in this study, indicated by the few women (10 in total) who did not provide an answer to the screening questions on symptoms of anhedonia and low mood. In addition, this study was limited by its cross-sectional nature which precluded direct comparisons between antenatal and postnatal survey results, particularly for ART-related factors, as the group of women on ART postnatally had more severe HIV disease than those on ART during pregnancy (which included women receiving ART for PMTCT only). Although approximately 80 % of HIV-positive pregnant women discontinue ART at delivery in Ukraine [27], almost half of the postnatal survey participants were on ART, reflecting differential follow-up and/or retention in HIV care of treated compared with untreated women. Evidence from other studies indicates that depression is associated with poorer antenatal self-care and an increased risk of loss to follow-up [5, 28–30]; the prevalence of depression among our survey group (who were all in contact with HIV services) may therefore not be generalizable to the overall population of pregnant and postnatal HIV-positive women in Ukraine.

The prevalence of depressive symptoms that we found (around 25 %), while indicating a substantial burden of poor mental health, is at the lower end of the prevalence range reported in previous studies among women living with HIV during pregnancy and the postnatal period [5]. Comparisons are problematic however due to heterogeneity of outcome measures and inclusion criteria used, and a lack of data from similar middle-income settings (previous studies have predominantly been from Africa and the US). Increased risk of depression among women with poorer social support, as indicated by our results among women without a cohabiting partner, is a common theme across settings [5, 31]. The greater anxieties and concerns around the use of ART among women reporting depressive symptoms may reflect low levels of knowledge and a more avoidant coping style in this group in contrast with an active coping style in which information and support is sought out, which is associated with lower depressive symptoms [32]. Depression has been linked with intrusiveness of symptoms among women living with HIV in a US study [33] and we found a significant association between depressive symptoms and self-reported severity of ART side-effects in pregnancy, which may indicate multiple risks for disengagement from treatment programmes postnatally in this group.

The World Mental Health Survey found that, in the general population in Ukraine, only 17.4 % of women with history of mood disorder had ever sought help from a medical professional, and among women with suicidal thoughts this proportion was only 28 % [4]. HIV-positive women’s unmet need for mental health services may be even greater due to barriers such as a lack of services integrated with HIV care, poorly developed outpatient infrastructure, out-of-pocket payments for psychiatric medication, and the double-stigma associated with HIV infection and mental health problems [4, 34]. In our study, we did not have information on participants’ history of depression, a possible risk factor for subsequent depressive episodes [31], or their previous access to mental health services. Referrals for psychological support were known to have been refused by three screen-positive women and more may have declined to attend, but the lack of functional linkages between HIV and psychological care in Ukraine and the anonymous nature of the study precluded a more detailed exploration of referral uptake.

Support services provided by non-governmental organisations (NGOs) such as support groups or peer counselling may help to maintain or improve psychological wellbeing of women living with HIV; however, these services are provided on an ad hoc basis with regional variability, and our results show that they were accessed predominantly after delivery. Women who have depressive symptoms before or during pregnancy are at increased risk of also experiencing these during the postnatal period [35, 36], a time of competing priorities and high risk for loss to follow-up, disengagement from care and declining ART adherence [37–40]. Routine screening and provision of support for depressive symptoms during pregnancy may provide an important opportunity to reduce the risk of loss to follow-up and poor outcomes after delivery.

To date, studies on interventions for depression for people living with HIV (which are predominantly from North America, and among men) have shown a range of strategies to be effective, particularly psychological interventions with a cognitive-behavioural component [41]. Cognitive-behavioural interventions aimed at increasing self-efficacy have been associated with reduced depressive symptoms and improved immunological and virological outcomes among women living with HIV in the US [42, 43], and could be of particular relevance to HIV-positive women in Ukraine given the associations we report between depressive symptoms and low ART-related self-efficacy. The ‘mothers2mothers’ programme in South Africa has shown a beneficial impact of peer support for women living with HIV on a range of psychosocial outcomes during pregnancy and postnatally [44]. Some women in our study were already receiving peer counselling; the role of this in protecting against depressive symptoms and related adverse maternal and child outcomes warrants further investigation. Further work is urgently needed to validate screening tools, evaluate interventions and improve access to mental health care for women living with HIV in Ukraine.

## Conclusions

Our results on the association between perinatal depressive symptoms and poor social support, anxieties about ART, low ART-self-efficacy and ART side effects highlight the multiple issues that women with depressive symptoms may face in optimising their own and their child’s health, and specific needs for counselling around the use of ART as well as for psychological support. There is an unmet need for proactive strategies to identify depressive symptoms and provide tailored treatment and support to this high-risk group of women living with HIV.

## EuroCoord Appendix

### EuroCoord Executive Board

Fiona Burns, University College London, UK; Geneviève Chêne (Chair), University of Bordeaux, France; Dominique Costagliola (Scientific Coordinator), Institut National de la Santé et de la Recherche Médicale, France; Carlo Giaquinto, Fondazione PENTA, Italy; Jesper Grarup, Region Hovedstaden, Denmark; Ole Kirk, Region Hovedstaden, Denmark; Laurence Meyer, Institut National de la Santé et de la Recherche Médicale, France; Heather Bailey, University College London, UK; Alain Volny Anne, European AIDS Treatment Group, France; Alex Panteleev, St. Petersburg City AIDS Centre, Russian Federation; Andrew Phillips, University College London, UK, Kholoud Porter, University College London, UK; Claire Thorne, University College London, UK.

### EuroCoord Council of Partners

Jean-Pierre Aboulker, Institut National de la Santé et de la Recherche Médicale, France; Jan Albert, Karolinska Institute, Sweden; Silvia Asandi , Romanian Angel Appeal Foundation, Romania; Geneviève Chêne, University of Bordeaux, France; Dominique Costagliola, INSERM, France; Antonella d’Arminio Monforte, ICoNA Foundation, Italy; Stéphane De Wit, St. Pierre University Hospital, Belgium; Peter Reiss, Stichting HIV Monitoring, Netherlands; Julia Del Amo, Instituto de Salud Carlos III, Spain; José Gatell, Fundació Privada Clínic per a la Recerca Bíomèdica, Spain; Carlo Giaquinto, Fondazione PENTA, Italy; Osamah Hamouda, Robert Koch Institut, Germany; Igor Karpov, University of Minsk, Belarus; Bruno Ledergerber, University of Zurich, Switzerland; Jens Lundgren, Region Hovedstaden, Denmark; Ruslan Malyuta (Chair), Perinatal Prevention of AIDS Initiative, Ukraine; Claus Møller, Cadpeople A/S, Denmark; Kholoud Porter, University College London, United Kingdom; Maria Prins, Academic Medical Centre, Netherlands; Aza Rakhmanova, St. Petersburg City AIDS Centre, Russian Federation; Jürgen Rockstroh, University of Bonn, Germany; Magda Rosinska, National Institute of Public Health, National Institute of Hygiene, Poland; Manjinder Sandhu, Genome Research Limited; Claire Thorne, University College London, UK; Giota Touloumi, National and Kapodistrian University of Athens, Greece; Alain Volny Anne, European AIDS Treatment Group, France.

### EuroCoord External Advisory Board

David Cooper, University of New South Wales, Australia; Nikos Dedes, Positive Voice, Greece; Kevin Fenton, Public Health England, USA; David Pizzuti, Gilead Sciences, USA; Marco Vitoria, World Health Organisation, Switzerland.

### EuroCoord Secretariat

Silvia Faggion, Fondazione PENTA, Italy; Lorraine Fradette, University College London, UK; Richard Frost, University College London, UK; Andrea Cartier, University College London, UK; Dorthe Raben, Region Hovedstaden, Denmark; Christine Schwimmer, University of Bordeaux, France; Martin Scott, UCL European Research & Innovation Office, UK.
